# Public health response to a tuberculosis exposure in a daycare: lessons on transmission risk and intervention

**DOI:** 10.1017/ash.2025.184

**Published:** 2025-05-13

**Authors:** Kari Neemann, Caleb Kuddes, Chad Wetzel, Alice I Sato, Justin Frederick

**Affiliations:** 1 Children’s Nebraska, Omaha, Nebraska, USA; 2 University of Nebraska Medical Center, Omaha, Nebraska, USA; 3 Douglas County Health Department, Omaha, Nebraska, USA

## Background

Over 9,600 cases of tuberculosis (TB) were diagnosed in the United States in 2023, a 16% increase over the previous year.^1^ When an individual is diagnosed with potentially contagious TB, public health agencies initiate a case investigation, which identifies individuals who have been in close contact with the index patient and provides them with recommendations for testing and, if necessary, treatment. The rapid identification of child contacts is particularly important as younger children have a higher risk of developing symptomatic tuberculosis disease.^2^ Additionally, the very young are more likely than other age groups to develop miliary, extrapulmonary, or meningeal TB acutely following infection.^2^ The CDC and WHO designate children <5 years of age exposed to TB as high-risk contacts who should be evaluated for window prophylaxis.^3,4^ Window prophylaxis is the administration of preventive antibiotic treatment to those who have been exposed to TB but have not yet developed symptoms or tested positive by immunologically based assays, and is recommended during the period between initial exposure and a follow-up test 8–10 weeks later to prevent the development of active TB.^3,4^ While it is known that the risk of TB infection increases with a longer exposure period,^5^ the duration of exposure time resulting in high-risk status has not been well described. Additionally, the density of infectious droplet nuclei in the air at the time of exposure and the ventilation capability of the environment also contribute to the risk of exposure.^3^

## Public health response to community TB exposure

This report details a public health response to a TB exposure at a Young Men’s Christian Association (YMCA) drop-in daycare center. The internationally infected source patient, without underlying health conditions, exhibited signs and symptoms of active pulmonary TB for three months before being diagnosed. This resulted in a potential exposure period totaling six months.^6^

Clinical findings of the index patient included a computed tomography scan showing a partially cavitary 7 mm right lung apex nodule, positive smear microscopy, GeneXpert MTB/RIF PCR, and AFB culture with *Mycobacterium tuberculosis* identified. The daycare consisted of a 1,473-square-foot room equipped with a standard HVAC system. The daycare utilized an electronic badge system to record the entry and exit of each child into the daycare center, allowing for highly accurate identification of contacts and exposure times.

**Figure 1.**
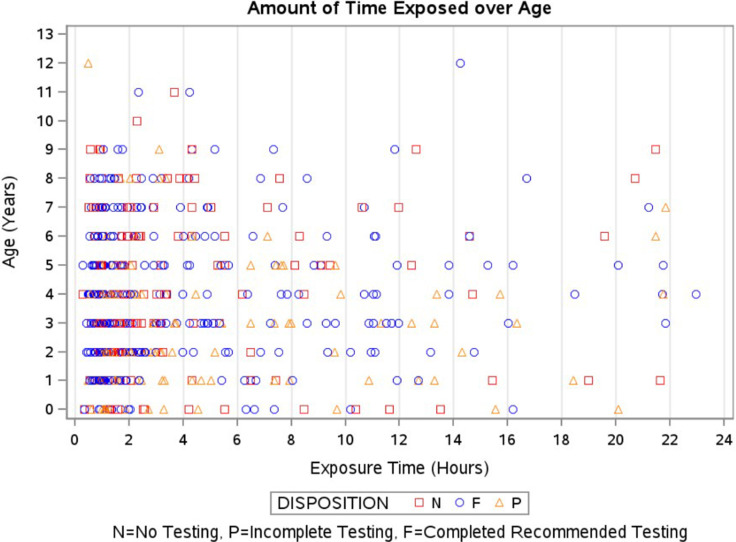
Cumulative time of tuberculosis exposure by age.

A total of 592 individuals had exposures related to the drop-in daycare, of which 359 were <5 years of age. Of those <5,211 had been exposed within the prior 10 weeks and were offered evaluation for window prophylaxis (Table 1). Cumulative exposure times ranged from 26 minutes to over 21 hours (Figure 1). The public health department, in consultation with the CDC’s Division of Tuberculosis Elimination, notified families with more than 30 minutes of documented exposure through multiple communication channels. These included emails (using contact information from YMCA membership profiles), phone calls, a public meeting, a press conference, and several press releases. Families were advised on patient-specific evaluations based on the last known exposure date. Individuals ≥5 years old or <5 years but more than 10 weeks from last exposure were offered TB testing at clinics set up at the YMCA (interferon- 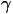 release assay [IGRA] ≥2 years or tuberculin skin test <2 years old). Individuals <5 years old and <10 weeks from exposure were referred to a TB Exposure Clinic developed in cooperation with the local children’s hospital where they had an exam performed by a provider, had chest x-ray and TB testing performed, and if indicated, initiated on prophylactic medication.

**Table 1.** Demographics of tuberculosis exposures at YMCA

		No testing/incomplete testing		Completed recommended testing		
		N	%	N	%	Total
Gender	Male	121	49.0	126	51.0	247
	Female	145	50.5	142	49.5	287
	Unknown	28	48.3	30	51.7	58
Race/Ethnicity	White, non-Hispanic	221	45.8	262	54.2	483
	Black, non-Hispanic	21	77.8	6	22.2	27
	Hispanic	23	59.0	16	41.0	39
	Asian	3	27.3	8	72.7	11
	Other	15	71.4	6	28.6	21
	Unknown	11	100.0	0	0.0	11
Age Group	0 – <24 Months	66	60.0	44	40.0	110
	24 – <60 Months	114	45.8	136	54.2	249
	5 – <10 Years	84	44.2	106	55.8	190
	10 – <15 Years	3	50.0	3	50.0	6
	15 – <19 Years	5	50.0	5	50.0	10
	19 Years and Older	22	81.5	5	18.5	27
Daycare Group	Attendee	267	48.1	288	51.9	555
	Staff	27	73.0	10	27.0	37
Exposure time Group	<2 Hours	120	44.0	153	56.0	273
	2 –12 Hours	122	51.3	116	48.7	238
	>12 Hours	25	56.8	19	43.2	44
	Unknown (Staff)	27	73.0	10	27.0	37
Window Prophylaxis Status	Recommended & Initiated (Prescribed)	74	62.2	45	37.8	119
	Recommended & Did Not Initiate	62	67.4	30	32.6	92
	Not Recommended	158	41.5	223	58.5	381
Total		294	49.7	298	50.3	592

A 1-step testing strategy was utilized for individuals with exposure ≥10 weeks from last contact and a 2-step testing strategy was utilized for those with <10 weeks from last exposure.^6^ Overall, 298 of 592 (50.3%) exposed individuals received recommended testing. Of the 211 individuals eligible for window prophylaxis (<5 years of age and <10 weeks from exposure), 107 of 211 (50.7%) completed initial testing, and 75 of 211 (35.5%) completed follow-up testing. Out of 211 individuals eligible for window prophylaxis, 92 (43.6%) initiated therapy. Notably, the acceptance rate increased to 93.7% (119 of 127) among those who were evaluated by a provider. As of this writing, none of the individuals identified in this community exposure have developed TB infection, and there are no known cases of TB disease among the non-household contacts.

## Discussion

The risk of developing TB disease among exposed infants and toddlers is high, necessitating prompt evaluation and consideration of window prophylaxis. This community exposure, involving 592 individuals, required a swift public health response. Within 72 hours of notification, the health department conducted an in-person informational session for families, held a press conference, issued a Health Alert Network Advisory, launched an online scheduler, and collaborated with the local children’s hospital for medical evaluations. To date, two household contacts have tested positive, resulting in an attack rate of 0.06%. No positive tests have been identified in non-household contacts, though only 49.6% of recommended testing was completed. Given that no child has been diagnosed with TB infection from this daycare exposure, it raises questions about whether the response scale was proportionate.

In 2013, Fang et al. reported a comparable community exposure in a Chinese high school, where the index patient went undiagnosed for three months. Contact tracing revealed that 61% (28/46) of students and 53% (9/17) of teachers in the primary classroom developed TB infection. Over the following year, three additional cases emerged among students not in close contact with the index patient, totaling 164 infected individuals out of 518 exposed. This included 4 confirmed TB cases, 20 probable cases, and 140 TB infections.^7^ Although exact exposure times were unknown, this case shows that prolonged exposure can increase TB risk, even without close contact. Both outbreaks involved undiagnosed infectious individuals, but the high school setting likely had more sustained interactions than the daycare, where exposure varied. The high school’s higher transmission rate (30% vs. 0.06%) suggests ventilation, exposure duration, and population density played key roles. Given China’s high burden of TB, infection may also have been acquired elsewhere.^8^ This highlights the importance of context-specific public health responses.

Conversely, limited exposure in a confined setting can also transmit TB. Kanamori et al. describe an 87-year-old woman with undiagnosed pulmonary TB confined to a disaster shelter in Japan in 2011, leading to a 20% prevalence of TB infection among co-evacuees over three days. Poor ventilation, as windows remained shut in cold weather, contributed.^9^ Our drop-in daycare had an HVAC ventilation system but no windows that opened outdoors, and the median exposure time was under five hours.

## Conclusion

The risk of TB infection is influenced by several variables. First, the density of infectious droplet nuclei produced by the source patient is challenging to quantify; second, the adequacy of ventilation in the exposure environment; and third, the duration of the exposure. Notably, 60% of our exposures involved young children, who are most vulnerable to disseminated TB disease. Although exposure time ranged from 29 minutes to over 21 hours, no transmission was observed in daycare-related contacts. This case provides evidence of low TB transmission risk among high-risk age groups in a classroom with exposure times under 24 hours, offering valuable insights for future outbreak investigations.

## Research Transparency and Reproducibility

NA.
